# Surface Properties of Squalene/Meibum Films and NMR Confirmation of Squalene in Tears

**DOI:** 10.3390/ijms160921813

**Published:** 2015-09-09

**Authors:** Slavyana Ivanova, Vesselin Tonchev, Norihiko Yokoi, Marta C. Yappert, Douglas Borchman, Georgi As. Georgiev

**Affiliations:** 1 Department of Biochemistry, Faculty of Biology, St. Kliment Ohridski University of Sofia, 1164 Sofia, Bulgaria; E-Mails: slavyana.plam.ivanova@gmail.com (S.I.); ggeorg@biofac.uni-sofia.bg (G.A.G.); 2 Institute of Physical Chemistry “R. Kaischew”-BAS, Phase Formation, Crystals and Amorphous Materials Department, 1113 Sofia, Bulgaria; E-Mail: vesselin.tonchev@gmail.com; 3 Department of Ophthalmology, Kyoto Prefectural University of Medicine, Kyoto 602-0841, Japan; E-Mail: nyokoi@koto.kpu-m.ac.jp; 4 Department of Chemistry, University of Louisville, Louisville, KY 40202, USA; E-Mail: mcyappert@louisville.edu; 5 Department of Ophthalmology and Visual Sciences, University of Louisville, Louisville, KY 40202, USA; 6 Biointerfaces and Biomaterials Laboratory, Department of Optics and Spectroscopy, Faculty of Physics, St. Kliment Ohridski University of Sofia, 1164 Sofia, Bulgaria

**Keywords:** Langmuir trough, meibum, NMR, sebum, squalene, tear film

## Abstract

Squalene (SQ) possesses a wide range of pharmacological activities (antioxidant, drug carrier, detoxifier, hydrating, emollient) that can be of benefit to the ocular surface. It can come in contact with human meibum (hMGS; the most abundant component of the tear film lipid layer) as an endogenous tear lipid or from exogenous sources as eyelid sebum or pharmaceuticals. The aims of this study were to determine (i) if SQ is in tear lipids and (ii) its influence on the surface properties of hMGS films. Heteronuclear single quantum correlation NMR confirmed 7 mol % SQ in Schirmer’s strips extracts. The properties of SQ/hMGS pseudo-binary films at the air/water interface were studied with Langmuir surface balance, stress-relaxation dilatational rheology and Brewster angle microscopy. SQ does not possess surfactant properties. When mixed with hMGS squalene (i) localized over the layers’ thinner regions and (ii) did not affect the film pressure at high compression. Therefore, tear SQ is unlikely to instigate dry eye, and SQ can be used as a safe and “inert” ingredient in formulations to protect against dry eye. The layering of SQ over the thinner film regions in addition to its pharmacological properties could contribute to the protection of the ocular surface.

## 1. Introduction

A thin film of lipids, the tear film lipid layer (TFLL), secreted mostly from the meibomian glands in the eyelids, covers the air/tear surface. TFLL is of key importance for ensuring the tear film (TF) low surface tension, high area-to-volume ratio and tangentially immobile air/tear interface opposing the outflow of aqueous tear in an open eye [1,2,3,4,5,6,7]. Human meibomian gland secretion (hMGS) also termed meibum is a composite lipid rich mixture that may contain up to 22 wt % non-lipid components (proteins, salts, polysachharides) [8]. Human meibomian lipids are composed of >90% non-polar lipids (primarily wax- and sterol esters and triacylglycerols) and <10% polar amphiphilic lipids ((*O*-acyl)-ω-hydroxy fatty acids (OAHFA) and some phospholipids) [1,2,9]. As hMGS is the main constituent of the TFLL, a considerable effort has been made to study its structure and properties at the air/water interface *in vitro* and *in vivo* and it was found to form a thick viscoelastic duplex film composed of a monomolecular layer of amphiphilic polar lipids at the aqueous surface and a generally unstructured lipophilic suspension, consisting of lipid lamellar-crystallite particulates immersed in a continuous liquid phase with no long-range order, located on top and facing the air [10,11,12,13]. However, there is growing evidence that, although hMGS lipids are the major ingredient (~90%–95%), meibum is not the only source of lipids in TFLL and admixtures of polar and non-polar lipids may be expected to come from other sources such as aqueous tears (e.g., from the lipocalin bound phospholipid pool) [14,15,16,17,18,19,20] or by eyelid sebum [21,22,23]. As even minor alterations in the composition of the lipid films can sometimes produce significant changes in their structure and properties, it is therefore important to study the interaction between such “exogenous” lipids and hMGS. This is even more relevant since more than 80% of the dry eye sufferers were recently found to report symptoms of meibomian gland dysfunction (MGD) a condition associated with qualitative and quantitative abnormalities in the TFLL [1]. Dry eye syndrome, DES, is characterized by decreased TF stability and the drying of the ocular surface leading to а painful sensation and the potential exposure of the eye to pathogens [3]. DES is a major ophthalmic public health disease that affects the quality of life and productivity of 10%–30% of the human population worldwide [24].

It is unclear whether eyelid sebum is an occasional contaminant or a normal supplement to the TFLL and cases for both can be made. On the eyelid, the glands of Zeis produce sebum and are very near the meibomian glands. Some mixing of meibum and sebum could occur as there is no physical boundary between both [6,25,26,27].

Upon blinking, the tear film becomes thicker [26] and the amount of lipid on the eyelid margin increases [28]. Although it is supposed that the accumulation of meibomian lipid on the eyelid margin should dilute the influx of skin lipids to the ocular surface [25], it was shown that lipophilic substances from the lower eyelid skin are able to reach the inferior tear meniscus supracutaneously and mix with the tear film lipid layer [29]. McDonald demonstrated [30] that skin lipid, delivered via rabbit’s whiskers, touched longitudinally to the cornea onto the surface of the tear film, disrupted its integrity along the touching line. However, later studies showed that mixing of sebum with hMGS films (with MGS being in excess to sebum) expands the meibomian layers and increases their surface activity [22]. Therefore, small amounts of sebum (or some of its constituents) could be beneficial for the TFLL to spread better and tolerate the high pressures of blinking.

**Figure 1:**
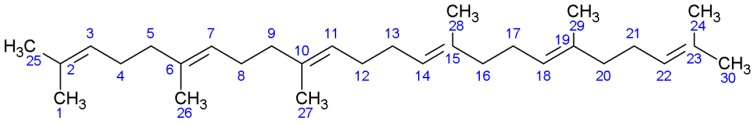
Structure of SQ. Numbering of carbons used throughout the text of this report.

A major (22%) compound of sebum is squalene (SQ) (Figure 1). Whether SQ is present in the TFLL as an intrinsic component of hMGS, or as a “contaminant” coming from the eyelid sebum, remains an open question. Older studies [31,32] reported up to 7% SQ in TFLL, but recent analysis by analysis by high pressure liquid chromatography-mass spectrometry [2] identified SQ only as a very minor component in aqueous tear samples. Borchman *et al.*, identified 1% SQ in hMGS and 4% to 6% SQ on the eyelid [21,23,33]. The amount of meibum on the eyelid and sebum on the forehead does not change with MGD [34], so the quality rather than quantity of meibum may contribute more to MGD. When SQ is placed on contact lenses, it inhibits the rate of evaporation [35]. Furthermore, SQ is of potential interest as an inclusion in pharmaceutical formulations as its delivery to the ocular surface is expected to exert antioxidant, drug carrier, detoxifier, hydrating, and emollient activities [36,37] and SQ could serve as a source of terpenoids found to be deficient in hMGS from MGD patients [38,39,40,41]. It was also shown in ^1^H NMR studies that the resonance at 5.2 ppm, which may belong to SQ, decreased with MGD [38,39,41]. However, once the intensity of the resonance is restored after azithromycin or doxycycline treatment, TF stability is recovered and patients no longer are afflicted by symptoms of dry eye. SQ may also affect the surface properties of the TFLL as it does to phospholipid films where condensation or expansion was observed depending on SQ content and film pressure [42,43,44].

The major aims of the current study were to examine the presence and concentration of SQ in human tear lipids with contemporary high resolution NMR spectroscopy and to study the effect of SQ on the surface properties of hMGS films. The presence of SQ in tears collected on Schirmer’s strips was probed by an inverse heteronuclear 2D NMR technique, heteronuclear single quantum coherence NMR spectroscopy (HSQC) that is one the most powerful methods available for tracing out the carbon skeleton of organic compounds [45]. The surface properties and structure of pseudo-binary films of SQ/hMGS were studied by Langmuir surface balance, stress-relaxation dilatational rheology and Brewster Angle Microscopy (BAM).

## 2. Results

### 2.1. NMR Studies

**Figure 2:**
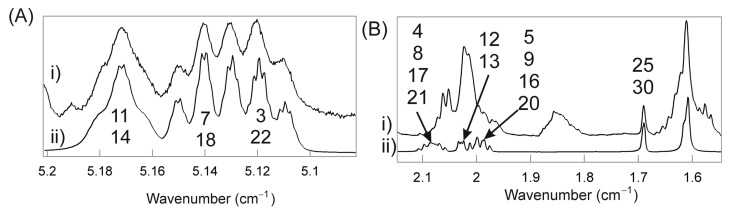
^1^H NMR spectra of tear lipids extracted from (i) Schirmer’s strip tear extracts (SSTE) or (ii) SQ. (**A**) =CH region; and (**B**) CH_3_, CH_2_ region. Numbers are the carbon numbers for SQ (Figure 1) assigned to the resonances.

**Figure 3:**
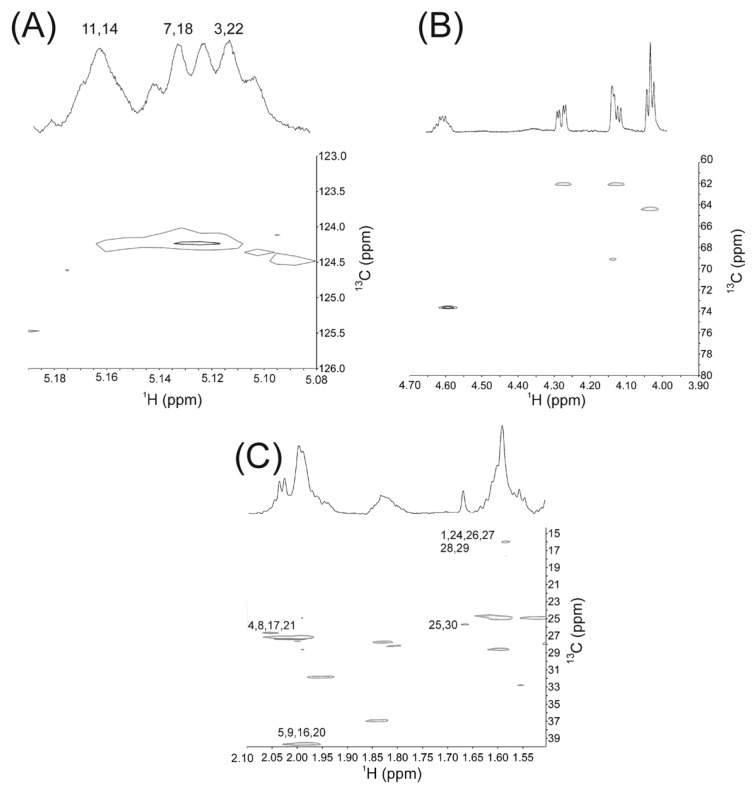
^1^H NMR spectra of tear lipid extracted from Shirmer’s strips atop the heteronuclear single quantum correlation (HSQC) spectra. Numbers correspond to the carbon numbers for SQ (Figure 1) assigned to the resonances. The chemical shifts are listed in Table 2. (**A**) CH resonance region; (**B**) Ester resonance region; and (**C**) CH_3_, CH_2_ resononance region.

The =CH resonance region accounts for six of the 50 protons of SQ (Figure 2Aii) that are characteristic of terpenoids (Figure 1). The close correspondence between the NMR resonance intensities (Table 1) and chemical shifts (Table 2) of human Schirmer’s strip tear extracts (SSTE) (Figure 2Ai,Bi) and SQ (Figure 2Aii,Bii) strongly suggests that the resonances in this region for SSTE are due to SQ. The HSQC spectra confirm the resonance assignments for this region of the ^1^H and ^13^C NMR spectra of SSTE (Figure 3, Table 2).

**Table 1:** Relative areas of ^1^H NMR resonances in the spectrum of Schirmer’s strip tear extracts (SSTE).

Squalene Moieties	Carbon Number	^1^H δ (ppm)	Experimental Relative Area *	Calculated Relative Area *
CH_3_	1, 24	1.61	Unresolved	0.12
	25, 30	1.67	0.12	0.12
	26, 29	1.61	Unresolved	0.12
	27, 28	1.62	Unresolved	0.12
CH_2_	4, 21	2.10	0.079	0.08
	5, 20	2.00	Unresolved	0.08
	8, 17	2.07	0.079	0.08
	9, 16	2.00	Unresolved	0.08
	12, 13	2.03	Unresolved	0.08
CH	3, 22	5.11	0.045	0.04
	7, 18	5.13	0.037	0.04
	11, 14	5.16	0.038	0.04

* The experimental relative area was determined by dividing the area of the proton resonances assigned to squalene in the spectrum of SSTE by the total areas of all the proton resonances assigned to squalene. Curve fitting was used for overlapping resonances. The calculated relative area was determined by dividing the number of protons by the total number of protons from the structure of squalene given in Figure 1.

**Table 2:** Confirmation of ^1^H and ^13^C NMR resonance assignments for squalene and SSTE.

Moiety	Carbon Number	^1^H δ (ppm) Squalene *	^1^H δ (ppm) SSTE	^13^C δ (ppm) Squalene *	^13^C δ (ppm) SSTE	HSQC Confirmation
CH_3_	1, 24	1.61	1.61	17.67	16.0	CH_3_ or CH
	25, 30	1.69	1.67	25.77	25.8	CH_3_ or CH
	26, 29	1.61	1.61	15.97	16.0	CH_3_ or CH
	27, 28	1.62	1.62	15.97	16.0	CH_3_ or CH
CH_2_	4, 21	2.09	2.10	26.79	27.2	CH_2_
	5, 20	2.00	2.00	39.74	39.6	CH_2_
	8, 17	2.09	2.07	26.79	27.2	CH_2_
	9, 16	2.00	2.00	39.74	39.6	CH_2_
	12, 13	2.03	2.03	28.37	28.3	CH_2_
CH	3, 22	5.11	5.11	124.31	124.25	CH_3_ or CH
	7, 18	5.13	5.13	124.15	124.25	CH_3_ or CH
	11, 14	5.17	5.16	124.19	124.25	CH_3_ or CH

* From citation [21,46].

From the intensities of the NMR ester resonances, we calculated the mole fractions of SQ, cholesteryl and wax esters and triglycerides in SSTE (Table 3) as described previously [9,23,33].

**Table 3:** Mole fractions of components of SSTE from a 61-year-old Caucasian male.

Moiety	Mole Fraction			
	SSTE *	Calculated 22 mol % Sebum Plus Meibum	Sebum ^†^	Meibum ^†^
Squalene	0.07	0.07	0.28 ± 0.06	0.01
Cholesteryl esters	0.38	0.27	0.03 ± 0.01	0.34
Triglycerides	0.10	0.09	0.38 ± 0.02	0.01
Wax esters	0.45	0.56	0.29 ± 0.05	0.64

* Experimental deviation based on standards is about 3%; and **^†^** from literature [22] *n* = 72.

**Figure 4:**
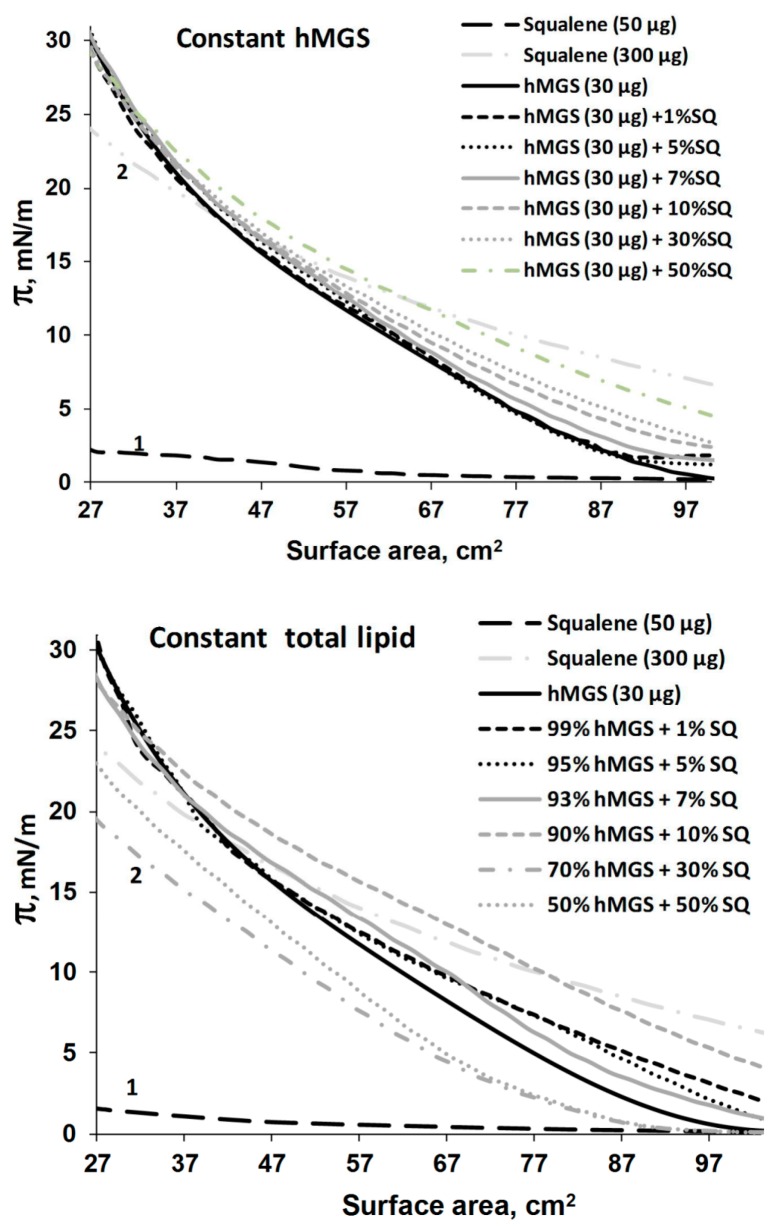
Compression π(A)-isotherms of hMGS, squalene (curve 1 and 2–50 and 300 µg SQ deposited at the trough surface respectively) and SQ/hMGS mixtures. The weight percentage of squalene is shown on the figure legends. **Upper panel** displays compression isotherms when the amount of total lipid (=hMGS + SQ) on the surface is kept constant and the hMGS/SQ ratio is varied within this fixed total lipid quantity; and **Bottom panel** shows compression isotherms when the hMGS amount is kept constant, and the addition of SQ increases the total lipid amount on the surface.

### 2.2. Langmuir Trough Studies

At 50 µg SQ deposited (*i.e.*, at 16.2 Å^2^ per molecule) over the trough area (Figure 4 isotherm 1) there was almost no increase of surface pressure at film compression, indicating that SQ has very limited surfactant properties. To register “full” isotherms (Figure 4, isotherm 2) reaching π = 25 mN/m at high compression, it was necessary to deposit 300 µg of SQ over the trough surface. This is a very large amount that corresponds to varying the apparent molecular area between 2.7 and 0.45 Å^2^ per molecule, *i.e.*, a stratified SQ multilayer at the air/water interface.

**Figure 5:**
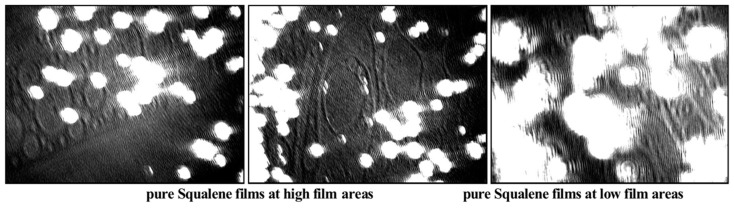
Characteristic UltraBAM images (720 µm × 400 µm) of squalene layers, which shows irregular and partial spreading of squalene with lens formation already at high film areas; at compression, the lenses aggregate and get thicker.

The characteristic UltraBAM images of pure SQ (≤50 µg SQ) (Figure 5) shows SQ to have irregular partial spreading at the air/water interface as expected for a hydrophobic nonpolar hydrocarbon. The large white spots are thick concentrated aggregates of lipid while the darker areas are thin layers consisting of much fewer lipid molecules.

The pseudo-binary hMGS/SQ films were obtained at two conditions: (i) at constant amount of total lipid (*i.e.*, the inclusion of SQ was accompanied by decrease in the hMGS quantity) and (ii) at constant amount of hMGS (when the addition of SQ increased the total lipid deposited at the trough surface).

The analysis of the π/A compression isotherms in the two cases revealed several trends (Figure 4). The presence of SQ resulted in increased π values at higher surface areas in both systems (fixed hMGS amount and constant total lipid).

When the amount of total lipid was kept constant, the raise of SQ content (in the range 1%–30%) resulted in modification of the shape of the π/A isotherms and in expansion (*i.e.*, higher surface area at identical π) of the mixed films at π ≤ 23 mN/m. The effect is clearly related with changes in the distribution of hMGS at the air/water surface as SQ has no surface activity at the concentrations used.

**Figure 6:**
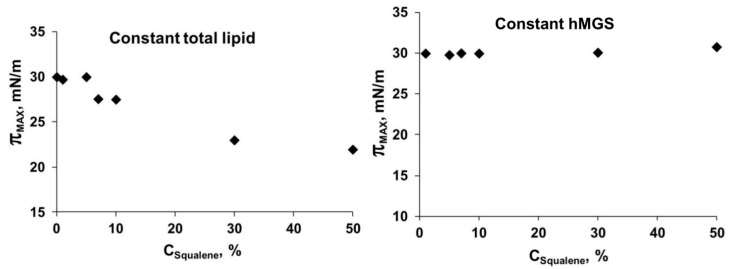
Dependence of the maximal surface pressure of mixed hMGS/SQ films on the concentration of squalene (in weight %). **Left panel** shows the dependence when the amount of total lipid (=hMGS + SQ) on the surface is kept constant and the hMGS/SQ ratio is varied within this fixed total lipid quantity; and **Right panel** shows the dependence when the hMGS amount is kept constant, and the addition of SQ increases the total lipid amount on the surface. The π_max_ value depends on the “end members” of the surface film. If, at the end of compression, the interface between the lipid multilayer and the aqueous subphase is enriched with molecules with surfactant properties (e.g., polar lipids) the π_max_ value is high. If, at the end of compression, the interface between the lipid multilayer and the aqueous subphase is poor on molecules with surfactant properties (e.g., polar lipids) the π_max_ value decreases.

The latter two points are in agreement with the “expanding effect” exerted by sebum (where SQ is major ingredient at 22% content) on human meibum films [22]. However, at further compression, the inclusion of SQ led to decreased capability of the hMGS/SQ layers to attain high surface pressure, π_max_ (Figure 6, left panel), at minimal film area (*i.e.*, at maximal degree of film contraction).

When the amount of hMGS was kept constant, the following features were observed: (i) the course of the π(A)-compression isotherms of the different hMGS/SQ layers tended to approach each other and at surface pressures π ≥ 15mN/m the π(A)-isotherms of films containing 1%–30% SQ almost overlapped and (ii) the maximal surface pressures (Figure 6, right panel) achieved at the end of compression by the pure hMGS films and the hMGS/SQ mixtures practically coincided.

**Figure 7:**
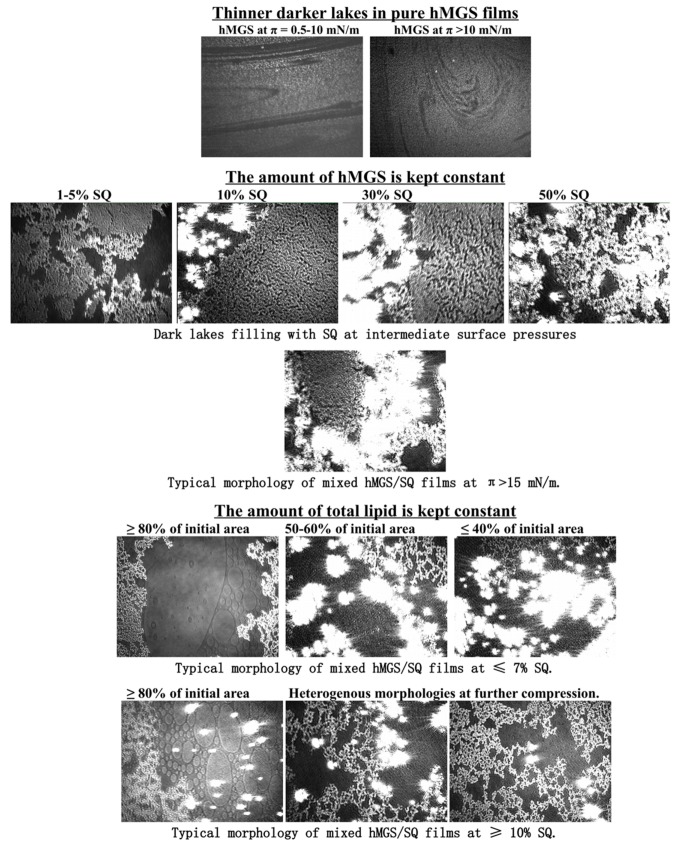
UltraBAM images (720 µm × 400 µm) of hMGS/SQ surface layers at various degrees of compression.

The UltraBAM images of hMGS, pure, and mixed with SQ (at constant total lipid or at fixed hMGS amount) are presented at Figure 7. Pure hMGS films showed characteristic rough structure, which was previously reported in detail [10,13] already at the lift off surface pressure consists of bright aggregates of multilayer thickness and dark regions of monolayer thickness (the content of the latter tends to diminish at further compression).

The addition of SQ in the pseudo-binary films altered the layers morphology in the course of the film compression. When the amount of hMGS was kept constant, thick bright islands were formed that overlay on the dark regions in the films, thus preventing the formation of the thinner areas in the meibomian films. In the case when the quantity of the total lipid was kept fixed, apart from the formation of the thick reflective islands, overall increase of the intensity (*i.e.*, the thickness) of the darker regions in the hMGS/SQ films was registered.

The similarity between the reflective islands observed in the hMGS/SQ layers and in the purely SQ films (Figure 5), together with the inability of SQ to alter the maximum surface pressure at fixed hMGS amount and the decrease of π_max_ with the raise in SQ content at constant total lipid allows for the suggestion that, in sufficiently densely packed surface films, SQ gets excluded from the air/water surface and localizes in the upper strata of the meibomian duplex film.

#### Analysis of Dilatation Rheology Properties of hMGS/SQ Films Evaluated in Stress/Relaxation Experiments

**Figure 8:**
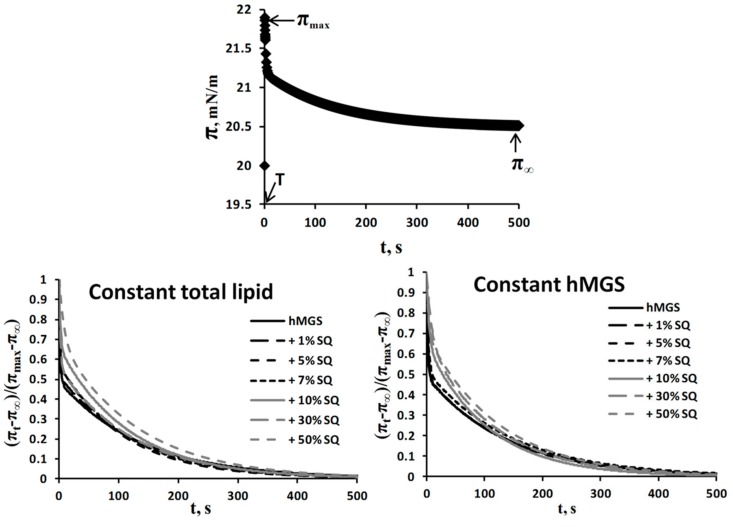
**Upper panel:** Typical stress-relaxation curve for film by equiweight meibum mixture (the subphase in the example is pure saline solution). The small compression deformation is completed in moment T and from this moment on the surface pressure starts to relax from a maximum value π_max_ to a new equilibrium value π_∞_. The surface pressure relaxation transient is analyzed by fitting with the equation of double exponential decay shown to chart (see Equation (1)); and **Bottom panels**: Surface pressure relaxation transients of hMGS/SQ films. The data are presented in the format required for further fitting by double exponential decay Equation (1) as dependence of (π_t_ − π_∞_)/(π_max_ − π_∞_) on *t*. The time axis is normalized to begin from the start of the relaxation (the moment T). Data from stress-relaxation experiments with all hMGS/SQ films are summarized in Table 4.

The surface pressure relaxation transients (Figure 8) were fitted to the two-exponential decay Equation (1). In the stress-relaxation experiments, hMGS/SQ film prior deformation was compressed to surface pressure 20 mN/m. After small instantaneous compression of the film is performed in order to establish a new equilibrium some molecular reorientation, adsorption/desorption, re-spreading, and structural rearrangement processes are necessary, which are not completed instantaneously. All processes on the scale of the short (<8 s) relaxation time, τ_fast_, can be described mainly by elasticity, while the slower processes, on the scale of the long (>100 s) relaxation time, τ_slow_, by viscosity. The values of *A*_fast_ and *A*_slow_ describe the contribution of the rapid “elastic” processes and of the slow “viscous” processes respectively to the total surface pressure relaxation. A typical transient of surface pressure relaxation is shown in the upper panel of Figure 8. The stress–relaxation data for meibomian film over saline subphase with varying amounts of SQ are shown in the lower panels of the figure.

For pure hMGS film, the contribution of the fast elastic relaxation to the total change of surface pressure was given by the constant *A*_fast_ > 0.51, and of the slow relaxation by *A*_slow_ < 0.49 (*i.e.*, the fast processes were slightly predominant in the rheological behavior of the film, see [10] for details). In contrast, with SQ, the values of *A*_slow_ were generally larger than those for *A*_fast_ as reflected by dropping of the ratio *A*_fast_/*A*_slow_ to <1 (Table 4).

**Table 4:** Values of the relaxation times, τ_fast_ and τ_slow_, and of the constants, *А*_fast_ and *А*_slow_, obtained by fitting the surface pressure relaxation transients (as the one shown on Figure 8) with the two-exponential decay equation by nonlinear regression; the fittings were with *R*^2^ ≥ 0.98. Results are summarized for meibomian films by an equiweight meibum mixture. Each data points is the mean of three measurements; S.D. is ±1.5%.

Film Composition		τ_fast_ (s)	*A*_fast_	τ_slow_ (s)	*A*_slow_	*A*_fast_/*A*_slow_
**Constant hMGS (30 µg); SQ Addition Increases Total Lipid**	hMGS	1.98	0.51	138.35	0.49	1.04
	+1% SQ	2.57	0.51	140.05	0.49	1.04
	+5% SQ	2.18	0.50	134.17	0.50	1
	+7% SQ	2.93	0.48	145.32	0.52	0.92
	+10% SQ	4.03	0.35	105.87	0.65	0.53
	+30% SQ	8.27	0.33	115.32	0.67	0.49
	+50% SQ	3.59	0.30	124.08	0.70	0.43
**Constant Total Lipid (30 µg); SQ Addition Is at the Expense of Decreasing hMGS Amount**	hMGS	1.98	0.51	138.35	0.49	1.04
	+1% SQ	1.05	0.48	128.03	0.52	0.92
	+5% SQ	1.37	0.43	109.16	0.57	0.75
	+7% SQ	1.43	0.43	115.83	0.57	0.75
	+10% SQ	2.39	0.34	116.30	0.66	0.52
	+30% SQ	2.19	0.44	123.29	0.56	0.78
	+50% SQ	7.53	0.30	129.55	0.70	0.43

## 3. Discussion

SQ-meibum interactions could be important to the functions of the TFLL. Using HSQC spectroscopy, we unambiguously confirmed the NMR spectrum of SQ in SSTE. The ^1^H resonances near 5.17 ppm correspond to the protons on carbons 11 and 14, and the resonance near 5.1 ppm is associated with the protons on carbons 3, 7, 22 and 18 (Figure 1, Table 1) [21]. Based on the lipid concentrations of sebum and meibum in Table 1, we calculate that about 22 mol % sebum with meibum would give a similar mole percent of moieties that we observe for SSTE. Of course, it should be kept in mind that, although widely used, Schirmer’s strips have certain limitations. Schirmer’s strips touch the ocular surface and might be contaminated with cellular debris. Thus, although the data on SSTE provide information on the lipids that may find their way into the human TF, a more noninvasive tear sampling technique, like capillary tubes [7], can allow researchers to unambiguously clarify the contribution of exogenous sources to the SQ amounts reported in tears.

Our Langmuir surface balance studies show that SQ localizes over the thinnest regions in the lipid layer and thus prevents the formation of thin regions within the surface films. When the amount of total lipids on the surface was kept constant, we found that the increase of SQ concentration resulted in film expansion at π ≤ 23 mN/m in agreement with the similar effect of a SQ-enriched sebum-meibomian layer [22] but with a decrease of the maximal surface pressure. The expansion caused by SQ would be expected and is in agreement with an infrared spectroscopic study that showed that the lipid hydrocarbon chain order in a mixture of meibum and squalene was 21% more disordered (more fluid) compared with meibum alone [22]. The phase transition temperature of the mixture of squalene and meibum was 5 °C lower compared with meibum alone [22]. More fluid, disordered hydrocarbon chains have more *gauche* rotamers and pack more loosely, with a greater area than ordered hydrocarbon chains.

When at identical hMGS/SQ ratios the amount of hMGS was kept constant (*i.e.*, the quantity of total lipids, hMGS plus SQ, was raised) the lift off area increased but at further compression after π ≥ 10–15 mN/m the isotherms of hMGS/SQ mixtures and of pure hMGS tended to overlap and the maximal surface pressure remained equal between all samples. The trend of the isotherms at high surface pressures and the value of π_max_ are determined by the composition of the “end members” of the surface film, *i.e.*, by the molecules which remained located at the interface with water at high compression instead of being squeezed in the subphase or in an upper strata of the lipid duplex layer [10]. We conclude from these observations that SQ molecules do not possess surfactant properties and, when mixed with hMGS, SQ localizes at the interface with the aqueous subphase only in expanded films with lower surface pressures. However, during compression at higher (>10–15 mN/m at fixed hMGS amount or at >23 mN/m at constant total lipid) surface pressures, the SQ molecules get pushed out from the interface with the aqueous subphase and migrate towards the upper strata of the hMGS film. As a consequence of this behavior, we conclude that SQ molecules do not contribute to the surface pressure of the hMGS films at physiological π (*i.e.*, at physiological lipid packing densities), but rather contribute to the thickness and to the normal structure of the hMGS multilayer as suggested by the formation of bright reflective aggregates in the pseudo-binary films in the presence of SQ (Figure 8). The layering of SQ on top of the thinner parts in the meibum film during compression can be partially explained with the hydrophobic nature of SQ molecules when more polar lipids of meibum are present which prefer to localize at the aqueous surface.

From the stress/relaxation transients of hMGS/SQ films we learn about the viscoelasticity of the film. The values of *A*_fast_ and *A*_slow_ describe the contribution of the rapid “elastic” processes and of the slow “viscous” processes respectively to the total surface pressure relaxation. A slightly higher value of *A*_fast_ compared with *A*_slow_ was observed for pure hMGS films (Table 4). The results agree with the viscoelastic properties of hMGS reported previously [10,47]. In contrast, with SQ, the values of *A*_slow_ were generally larger than those for *A*_fast_, which means that, when the amount of SQ increased, the slow “viscous” contributions to the rheological properties of hMGS prevailed over the fast “elastic” contributions (Table 4). As the melting temperature of SQ is −75 °C, SQ is in a disordered state at physiological temperature. It is known that in disordered lipid films, the contribution of the elastic processes to the rheology properties decreases, while the one of the viscous processes increases [48]. Viscoelasticity of meibum was also found to decrease when the lipid became more disordered [10,47]. As our Langmuir surface balance results showed, the relaxation should take place in pseudo-binary films of constituents with limited miscibility: the wax-enriched hMGS and SQ that tends to overlay on top as thick reflective aggregates. Probably, the completion of the multiple processes involved in the relaxation (*i.e.*, all the molecular reorientations, adsorption/desorption, re-spreading, and structural rearrangements necessary to reach new equilibrium after the step-like instantaneous deformation is ceased) will take longer in such composite pseudo-binary lipid layers of separated compounds each with its own relaxation rate and loosely synchronized between each other in comparison with more uniformly miscible systems. In addition, in the case of constant total lipid amount at the surface, the increase of SQ content is at the expense of the decrease of hMGS quantity, which, in turn, results in diminished number of amphiphilic meibomian “polar” lipids at the interface which are of crucial importance for the spreading and viscoelasticity of the tear film lipid layer.

The Langmuir surface balance studies of the interaction of target molecules (here SQ) with MGS films gained popularity in recent years in relation to the simultaneous efforts of several groups [4,10,12,13]. Such experiments allow the phenomena to be probed in precisely defined simplified conditions. That feature is both the major advantage and limitation of this *in vitro* approach. From one side, it is possible to see the interplay between two components “pure” without signal interference due to the simultaneous interaction of the tested molecule with all other constituents of the TF (aqueous tears, glycocalyx *etc.*). However, the simplification of the *in vitro* system inevitably bears the risk of overlooking the contribution of other tear constituents to the ingredients’ impact on TF *in vivo*. A complimentary step-by-step approach is possible in which the *in vitro* system is gradually enriched with compounds to finally closely emulate the physiological environment. Although laborious, such methods enable researchers to get an insight to the individual contribution of each tear component to the impact of an exogenous ingredient on the material properties of the TF. The molecule used in this study, SQ, is extremely lipophilic and water insoluble. Therefore, once it reaches the ocular surface, it is expected to distribute exclusively in the TFLL and, in particular, into its stratum of meibomian nonpolar lipids. Therefore, the SQ/hMGS interaction is relevant to study. Control experiments with axisymmetric drop shape analysis (as previously described in [48]) of SQ/whole tear samples revealed an identical trend. SQ spread on the surface of tear drops at 0.2 µg/cm^2^ (the maximum surface concentration of SQ in the SQ/hMGS pseudo-binary films) did not affect the interfacial properties of whole tears (data not shown). Thus, it can be reasonably assumed that supplementation of SQ to the ocular surface will not alter TFLL surface properties.

The limited effect of SQ on the surface properties of meibum indicates that squalene can be readily implemented as a safe and “inert” ingredient in pharmaceutical formulations that will not alter the stability of the tear film. At the same time, SQ ability to solubilize drug molecules in lipid emulsions is widely noted [36,37] and, in future, SQ can be utilized as a carrier in ocular surface drug delivery. Furthermore, SQ can exert its own positive effects (antioxidant, detoxifier, hydrating, and emollient) at the ocular surface [36,37].

In conclusion, SQ is a component of SSTE. SQ does not possess surfactant properties and, when mixed with hMGS, it does not contribute to the surface pressure of the hMGS films at physiological π. If the eye is kept open long enough (>20 s), evaporation causes the tear film to break up forming deleterious dry regions on the surface of the cornea without a visible aqueous or lipid layer. The layering of SQ over the thinner lipid layer regions in addition to its antioxidant, antibacterial, and anti-inflammatory properties could contribute to the protection of the ocular surface.

## 4. Experimental Section

### 4.1. Collection and Processing of Human Meibum for Langmuir Trough Analysis

Written, informed consent was obtained from all donors. Protocols and procedures performed at the University of Louisville were reviewed by the University of Louisville Institutional Review Board. For the Langmuir surface balance studies, the samples were collected from the eyelid margin with a platinum spatula, weighed, and dissolved in chloroform to a pooled stock solution with a concentration of 1 mg lipid/mL. hMGS samples were collected from healthy volunteers working in the laboratory, three females (25–43 years old) and one male (33 years old). The characteristic reversible π(A)-isocycle and multilayer structure of hMGS films was similar to that published in Figure 3 of citation [49]. Collection of hMGS for Langmuir trough and ultraBAM Brewster angle microscopy (BAM) and Langmuir surface balance experiments were essentially identical to those reported in our previous publication [49]. The experiments were performed with equiweight hMGS, which in our experience is well representative for the material properties of meibomian lipids collected from a variety of healthy volunteers. The standard deviation between the measured compression isotherms is less than 2%.

### 4.2. Collection of Tears for NMR Spectroscopic Analysis

Samples were collected from a Caucasian male, 61 years old, with no signs or symptoms of dry eye. To collect tears, unmarked Shirmer strips (Alcon Inc., Fort Worth, TX, USA) were placed inside the lower eyelid (inferior fornix) near the center of each eye for 5 min and then removed. Tears were collected once a day at 10 a.m. for 24 days. Care was taken not to touch the lower third of the strips, which were cut and placed into a glass scintillation vial and stored in the dark, under argon at −20 °C. The samples were sonicated with 5 mL methanol under an atmosphere of argon gas in an ultrasonic bath (Branson 1510, Branson Ultrasonics, Danbury, CT, USA) for 10 min. The strips were removed from the vial and placed into another vial containing 5 mL of chloroform. The sample was again sonicated. The strips were removed and the chloroform from the extraction was mixed with the methanol from the previous extraction and evaporated together under a stream of argon gas. The sample was lyophilized for 1 h to remove trace solvent and weighed. CDCl_3_ (500 µL) was added to the 5.75 mg of dried extract. The sample was sonicated under an atmosphere of argon gas in an ultrasonic bath (Branson 1510, Branson Ultrasonics, Danbury, CT, USA) for 10 min and placed into an NMR tube for spectral measurement. CDCl_3_, SQ and tetramethylsilane (TMS) used in spectroscopy measurements were obtained from Sigma-Aldrich (St. Louis, MO, USA).

### 4.3. NMR Spectral Measurements

Spectral data were acquired using a Varian VNMR 700 MHz NMR spectrometer (Varian, Lexington, MA, USA) equipped with a 5 mm ^1^H{^13^C/^15^N} ^13^C-enhanced cold probe (Varian, Palo Alto, CA, USA). Spectra were acquired with a minimum of 250 scans, 45° pulse width, and a relaxation delay of 1.000 s. All spectra were obtained at 25 °C. HSQC was performed using 512 increments with 16 scans per increment, 45 ° pulse width, and a relaxation delay of 1.000 s, mixing time of 0.080 s, and a one-bond coupling constant threshold of 140 Hz. Spectral acquisition took about 10 h. Spectra were analyzed using MestReNova software, version 7.1.2-10008 (Mestrelab Research S.L., Santiago de Compostela, Spain). The TMS resonance was set to 0 ppm. Commercial software (GRAMS 386; Galactic Industries Corp., Salem, NH, USA) was used for spectral deconvolution and curve fitting. The area of each band was used for the quantification of lipid composition [9,23].

### 4.4. Langmuir Trough Studies

#### 4.4.1. Compression Isotherms

Surface pressure–area (π-A) isotherms were measured using a computer-controlled Langmuir surface balance (Kibron, Helsinki, Finland) equipped with an automated trough (µTrough XS, area 135 cm^2^, volume 20 mL) by the Wilhelmy wire probe method (instrumental accuracy 0.01 mN/m). The trough subphase consists of physiological saline solution. Human MGS or hMGS with SQ, both dissolved in chloroform, was spread (30 µL of 1 mg/mL) over the air/saline solution interface with a microsyringe (Hamilton Co., Reno, NV, USA). The maximal weight % of SQ used in the studies was 50%. Higher concentrations of SQ are not physiologically relevant and result in the loss of reproducibility in π(A)-isotherms. An acrylic cover was put over the trough to protect the surface from dust and to suppress subphase evaporation. After 15 min to allow chloroform evaporation, film area compression was started using two symmetrically moving barriers. Fast dynamic compression–expansion isocycling of the film area was performed with the maximal possible barrier’s rate (80 cm^2^/min) at which there was no leakage of the film. Ten consecutive cycles were performed with each sample. Normally, between the first and third cycles, the π(A) loops remained constant and those π(A) isocycles are presented and analyzed. All isotherms were repeated at least three times; the difference between the repetitions did not exceed 2%. Isotherm hysteresis was minimal between repeated isotherms of meibum films at a single temperature and that is why only compression isotherms are presented in subsequently. The experiments were performed at 34 °C. The morphology of the films was observed by UltraBAM (Accurion Gmbh, Germany) [50].

#### 4.4.2. Stress-Relaxation Studies via the Small Deformations Method

In order to gather information about the viscoelastic properties of meibum films, pure and with SQ, we monitored the relaxation of the surface pressure after a small rapid compression deformation was applied to the surface film. Prior to deformation, the film was compressed to surface pressure of 20 mN/m. Then the lipid film was instantaneously and slightly contracted with a small area perturbation, ∆*A*/*A*o = 5% ± 1% (where *A*o is the initial area of the film, and ∆*A* is the area change). Then the relaxation of the surface pressure (see Figure 8 for details) was registered; for the samples studied, it usually took ≤500 s for complete relaxation. The surface pressure relaxation kinetics is described by the following equation [48]:

(1) $$\frac{\pi (t)-\pi_{\infty }}{\pi_{\text{max}}-\pi_{\infty }}=A_{\text{fast}}\text{exp}(t/\tau_{\text{fast}})+A_{\text{slow}}\text{exp}(t/\tau_{\text{slow}})$$

where π(*t*) is the surface pressure at a given time *t*; π_max_ is the maximal surface pressure at the start of the relaxation, immediately after the compression is performed; π_∞_ is the equilibrium surface pressure after completion of the relaxation; τ_fast_ and τ_slow_ are relaxation times for rapid and slow processes which take part in the total relaxation process; *А*_fast_ and *А*_slow_ are constants which reflect the contribution of the fast and slow relaxation times, respectively, to the total surface pressure change.
